# Development and description of measurement properties of an instrument to assess treatment burden among patients with multiple chronic conditions

**DOI:** 10.1186/1741-7015-10-68

**Published:** 2012-07-04

**Authors:** Viet-Thi Tran, Victor M Montori, David T Eton, Dan Baruch, Bruno Falissard, Philippe Ravaud

**Affiliations:** 1 Université Paris Descartes, Faculté de Médecine, Paris, France; 2 INSERM U738, Paris, France; 3 Division of Health Care and Policy Research, Department of Health Sciences Research and Knowledge and Evaluation Research Unit, Mayo Clinic, Rochester, MN, USA; 4 Université Paris Denis-Diderot, Faculté de Médecine, Paris, France; 5 INSERM U669, Paris, France; 6 Université Paris Sud, Paris, France; 7 Department of Epidemiology, Columbia University Mailman School of Public Health, New York, NY, USA

**Keywords:** chronic disease/therapy, patient participation, physician-patient relations, quality of life, questionnaires, workload

## Abstract

**Background:**

Patients experience an increasing treatment burden related to everything they do to take care of their health: visits to the doctor, medical tests, treatment management and lifestyle changes. This treatment burden could affect treatment adherence, quality of life and outcomes. We aimed to develop and validate an instrument for measuring treatment burden for patients with multiple chronic conditions.

**Methods:**

Items were derived from a literature review and qualitative semistructured interviews with patients. The instrument was then validated in a sample of patients with chronic conditions recruited in hospitals and general practitioner clinics in France. Factor analysis was used to examine the questionnaire structure. Construct validity was studied by the relationships between the instrument's global score, the Treatment Satisfaction Questionnaire for Medication (TSQM) scores and the complexity of treatment as assessed by patients and physicians. Agreement between patients and physicians was appraised. Reliability was determined by a test-retest method.

**Results:**

A sample of 502 patients completed the Treatment Burden Questionnaire (TBQ), which consisted of 7 items (2 of which had 4 subitems) defined after 22 interviews with patients. The questionnaire showed a unidimensional structure. The Cronbach's α was 0.89. The instrument's global score was negatively correlated with TSQM scores (r_s _= -0.41 to -0.53) and positively correlated with the complexity of treatment (r_s _= 0.16 to 0.40). Agreement between patients and physicians (n = 396) was weak (intraclass correlation coefficient 0.38 (95% confidence interval 0.29 to 0.47)). Reliability of the retest (n = 211 patients) was 0.76 (0.67 to 0.83).

**Conclusions:**

This study provides the first valid and reliable instrument assessing the treatment burden for patients across any disease or treatment context. This instrument could help in the development of treatment strategies that are both efficient and acceptable for patients.

## Background

Chronic diseases are the leading cause of mortality in the world, representing more than 36 million deaths in 2008 [1]. About 45% of the population and 88% of people older than 65 years have at least one chronic condition. The prevalence of chronic diseases continues to increase: in 2020, nearly 50% of the US population will have at least one chronic condition [2]. Therefore, the challenge for physicians has switched from curing acute illnesses to managing multiple chronic conditions. However, illnesses are still the primary focus of medical care [3] and many clinical practice guidelines focus on single conditions. For example, a physician following extant guidelines could prescribe up to 12 medications for a patient with osteoporosis, osteoarthritis, type 2 diabetes mellitus, hypertension, and chronic obstructive pulmonary disease [4].

Being a patient implies more investment of time and effort than just taking medicines. It also involves drug management, self-monitoring, visits to the doctor, laboratory tests and changes of lifestyle. For example, patients with type 2 diabetes controlled by oral agents could spend 143 minutes daily in recommended self-care [5]. This workload can affect quality of life as severely as the illness itself, and patients rate this treatment burden equal to that of diabetic neuropathy or nephropathy [6].

Treatment burden can be defined as the impact of health care on patients' functioning and well-being, apart from specific treatment side effects [7,8]. It takes into account everything patients do to take care of their health: visits to the doctor, medical tests, treatment management, and lifestyle changes. Treatment burden is associated, independently of illnesses, with adherence to therapeutic care [9,10] and could affect hospitalization [11] and survival rates [12].

Minimally disruptive medicine seeks to tailor treatment to the contexts of patients by integrating the notion of treatment burden in their care [13]. Therefore, caregivers need tools to establish the weight of the treatment burden. Many instruments assess treatment burden for specific conditions [14-18], but none has been developed to assess this burden globally across multiple chronic diseases. Because the treatment burden grows from the combination of chronic diseases, only an instrument that assesses it globally could help clinicians and researchers develop effective therapeutic programs that minimize the treatment workload [13].

In the present work, we aimed to develop a measure of treatment burden for patients with at least one chronic condition. This measure should be of use in daily clinical practice and in clinical research.

## Methods

We used a multistep method to develop a tool to measure the treatment burden of chronic diseases [19,20] following the quality criteria proposed in the literature [21].

### Stage 1: elaboration of the questionnaire

The objective of the instrument was to capture the perception of treatment burden of patients as 'the work of being a patient' dealing with increasingly complex treatment regimens [13], that is, the impact of the workload of healthcare on a patient's well-being and functioning.

We searched MEDLINE via PubMed for literature on treatment burden and existing questionnaires assessing it in specific diseases. We found no instrument appraising the treatment burden globally. Treatment burden was often assessed only as a subscale of specific disease scales [14-17] and thus was considered only for the regimen associated with a particular condition. Items often focused on drug intake, adherence to care and convenience of use.

Using this literature review, three members of the team who had experience in the care of patients with chronic diseases (V-TT, BF, PR) highlighted possible relevant topics to capture the aspects of the workload of healthcare that could affect a patient's life. These topics were the burden associated with taking medicines, self-surveillance, laboratory tests, doctor visits, need for organization, administrative tasks, following advice on diet and physical exercise and social impact of the treatment. According to the conceptual model of our instrument, we chose not to include other consequences of the treatment such as treatment side effects.

In addition, because our instrument was elaborated in France and administered to French patients, we did not take into account the financial burden of treatment, because our national public health insurance program guarantees healthcare free of charge for patients with chronic conditions.

We recruited a convenience sample of 22 patients with at least 1 chronic condition from the department of internal medicine of Hospital Pitié-Salpetrière and a general practitioner clinic in Paris in April 2011 (Additional file 1, Appendix 1). These two settings involved patients with various chronic conditions, requiring primary, secondary and tertiary care. During semistructured interviews, we presented the concept of treatment burden to patients and asked them about their diseases, their treatment and the burden of treatment, with open-ended questions: 'Could you tell us about your health problems?' 'Could you tell us about what you have to do to take care of your health?' 'What aspects of your care have the most impact on your life?' Then, we asked them about the burden associated with the different topics highlighted earlier by asking them (1) to rate each of these items, (2) to explain why they would rate it like that and (3) if they found the item relevant in the assessment of treatment burden generally. Finally, we asked patients, if other aspects of the workload of healthcare bothered them. As a result of these interviews, examples were added to the items, and we added one item 'Frequent healthcare reminds me of my health problems' to the questionnaire.

The resulting questionnaire consisted of seven items (two of which had four subitems), formed by an introductory sentence with examples, followed by a rating scale ranging from 0 to 10 with numbers placed under boxes and labeled end anchors ('No burden' and 'Considerable burden') [22-24].

A group of ten physicians (two methodologists, three general practitioners, two internists, one cardiologist, one pneumologist, one diabetologist) with experience in the care of patients with chronic conditions, some of whom had experience in questionnaire development, reviewed the clarity and wording of the items. All physicians agreed that, on the surface, items appeared to be measuring what they actually were and that the instrument achieved face validity.

### Stage 2: measurement properties of the instrument

The measurement properties of the questionnaire were assessed by four steps: (1) reduction of the number of items, (2) assessment of factorial validity, (3) assessment of construct validity and (4) assessment of reliability.

We recruited consecutive patients from six teaching hospitals of the Assistance-Publique Hôpitaux de Paris and eight general practitioner clinics in Paris to validate the questionnaire. Patients were eligible if they were 18 years or older, were able to complete a consent form and had at least one condition requiring medical follow-up for at least 6 months. Patients with cognitive impairment that could interfere with understanding the questionnaire were excluded. All patients provided written informed consent to be in the study.

Reducing the number of items was based on (1) a floor effect, considered present if more than 15% of respondents had the lowest score [21]; (2) the relevance of the items, assessed by the number of answers for which patients checked 'Does not apply'; and (3) item redundancy, suspected when interitem correlations by Spearman's correlation coefficient were > 0.80 [19]. Items were eliminated after discussion among three investigators (V-TT, BF, PR).

Answers to the questionnaire were aggregated in a global score by summing the item responses. 'Does not apply' or missing answers were considered the lowest possible score (0) because we considered that a patient not concerned by a domain of the treatment burden had no burden for that domain.

Factorial validity was assessed by determining the dimensional structure of the questionnaire by use of factor analysis. Scree plots were used to visualize a break between factors with large and small Eigenvalues. Factors that appeared before the horizontal break were assumed to be meaningful. Internal consistency was assessed by Cronbach's α [25] and was considered acceptable between 0.70 and 0.95 [26].

Construct validity was obtained by confirming two constructs theorized on the treatment burden [27]. First, we hypothesized a negative correlation between treatment burden, defined as the work of dealing with complex treatment regimens, and treatment satisfaction, defined as the balance between expectations about the treatment, side effects, convenience of use, and perceived efficacy. Treatment satisfaction was assessed by the Treatment Satisfaction Questionnaire for Medication (TSQM), an 11-item questionnaire validated in a population with diverse chronic conditions, measuring patient satisfaction with various medications designed to treat, control or prevent a wide variety of medical conditions [28,29]. TSQM scores range from 0 to 100 and measure patient satisfaction with the treatment's effectiveness, side effects, convenience and globally. Correlations were expected to be higher between our instrument and the TSQM convenience score because some items overlapped. Second, we assumed a positive correlation between the patient evaluation of the treatment burden and treatment workload evaluated by items on (1) drug intake (number of tablets, injections and intakes per day); (2) medical follow-up (number of different physicians, medical appointments per month and hospitalizations per year); and (3) daily time spent on self-care. The correlations between the global questionnaire score, the TSQM scores and treatment workload variables were assessed by Spearman correlation coefficient (r_s_) and considered high with r_s _> 0.50 and moderate with r_s _0.35 to 0.50 [30]. Wilcoxon and Kruskal-Wallis tests were used to compare measurements for qualitative variables across groups. A *P *value < 0.05 was considered statistically significant. We used linear regression analyses to examine variables that predicted the global questionnaire score. Relationships were characterized with beta coefficients, standard errors, and percent variance explained (adjusted R^2^) within these models. Heteroskedasticity was corrected by the method described by Greene *et al. *[31].

Description of our sample was completed by clustering homogenous groups of patients depending on the similarity of their response patterns to the Treatment burden questionnaire and analysis of treatment workload variables in each cluster of patients. Clustering involved a hierarchical ascendant classification with a Ward's distance method [32]. The number of clusters was determined so as to have a minimal sample of 100 patients. Stability of clustering was assessed by a twofold crossvalidation method.

We compared the patient's self-evaluation of treatment burden with an evaluation by their physician and by an informal caregiver using the same questionnaire adapted for heteroevaluation. Physicians and informal caregivers were asked to make the best estimate of the patient's treatment burden from their perspective.

Reliability of the instrument was determined by a test-retest method. Patients completed the new instrument twice: at baseline and after 2 weeks or 1 month. Reliability was assessed by the intraclass correlation coefficient (ICC) for agreement [33]. The 95% confidence intervals (95% CIs) were determined by a bootstrap method. Agreement was considered acceptable with ICC > 0.60 [27,34]. Agreement was represented by Bland and Altman plots, which represent the differences between two measurements against the means of the two measurements [35].

Statistical analyses involved use of SAS v. 9.2 (SAS Institute, Cary, NC, USA) and R v. 2.13.1 http://www.r-project.org/. This study was approved by the Institutional Review Board of Hospital Bichat (IRB: 00006477).

## Results

In total, 502 patients (mean age 59.3 (± 17) years; 266 women (53.1%)) were included to validate the questionnaire from April 2011 to September 2011 in France; 257 were inpatients (51.2%) and 300 reported a symptomatic disease (62.6%) (Table 1). Self-reported main chronic conditions ranged from diabetes (16.5%) to cancers (6.9%) and included well controlled psychiatric illnesses (1.6%).

**Table 1:** Demographic and clinical characteristics of patients (n = 502)

Patient characteristics	Value	Missing data
Age, years (range)	60 (19 to 94)	2
Female sex, no. (%)	266 (53.1%)	1
Marital status, no. (%):		13
Married	216 (44.2%)	
Live-in partner	38 (7.8%)	
Single/separated	171 (35.0%)	
Widowed	64 (13.1%)	
Highest education level, no. (%):		44
No diploma/primary school	85 (18.6%)	
Secondary/high school	195 (42.6%)	
College	178 (38.9%)	
Inpatient, no. (%)	257 (51.2%)	
Duration of disease, years (range)	10 (0 to 91)	33
Presence of daily symptoms, no. (%)	300 (62.6%)	23
Need for assistance, no. (%)	132 (26.4%)	2
Hospitalizations during the last 12 months, no. (%)	0 (0 to 15)	40
Medical appointments/month, no. (%)	1 (0 to 30)	16
Different physicians, no. (%)	2 (0 to 10)	18
Tablets/day, no. (%)	4 (0 to 30)	14
Drug intakes/day, no. (%)	2 (0 to 6)	26
Injections/day, no. (%)	0 (0 to 8)	78
Diet, no. (%)	198 (40.3%)	11
Physical therapy, no. (%)	113 (22.9%)	9
Oxygen therapy, no. (%)	22 (4.4%)	4
Need for a specific organization for daily care, no. (%)	338 (67.3%)	
Time needed to organize drugs/week^a^	60 min (0 to 21 h)	
Need for self-monitoring, no. (%)	168 (33.47%)	
Time needed for self-monitoring/week^a^	60 min (0 to 12 h)	
Presence of side effects, no. (%)	168 (36.3%)	39
Main chronic condition, no. (%):		11
Diabetes	81 (16.5%)	
Rheumatologic diseases	59 (12.0%)	
High blood pressure and dyslipidemia	44 (9.0%)	
Systemic diseases	43 (8.8%)	
Pulmonary diseases (other than asthma)	40 (8.1%)	
Heart diseases	37 (7.5%)	
Asthma	37 (7.5%)	
Cancers and hematological malignancy	34 (6.9%)	
HIV infection	19 (3.9%)	
Arterial or venous thrombosis	17 (3.5%)	
Other diseases^b^	80 (16.3%)	

^a^Median time needed for concerned patients (self-reported)

^b^Other diseases include diseases of the digestive system, psychiatric diseases, allergies, non-malignant hemopathy, neurological diseases, sequelae of injury, and endocrine diseases (other than diabetes).

During item reduction, we eliminated the subitem 'The conditions to store your medications (in your refrigerator etc.)' because a large number of patients responded 'Does not apply' (51.6%) and it had a large floor effect (64.0%) (Additional file 2, Appendix 2). Therefore, the final version of the questionnaire, the Treatment Burden Questionnaire (TBQ), consisted of seven items (two of which had four subitems) (Table 2).

**Table 2:** Items of the final Treatment Burden Questionnaire

**Item no**.	Item
1A	The taste, shape or size of your tablets and/or the inconvenience caused by your injections (for example, pain, bleeding, scars)

1B	The number of times you have to take your medication every day

1C	The things you do to remind yourself to take your daily medication and/or to manage your treatment when you are not at home

1D	The specific conditions when taking your medication (for example, taking it at a specific time of the day or meal, not being able to do certain things after taking them like driving or lying down)

2A	Lab tests and other exams (frequency, time spent and inconvenience of these exams)

2B	Self-monitoring (for example, taking your blood pressure or measuring your blood sugar yourself: frequency, time spent and inconvenience of this surveillance)

2C	Doctors visits (frequency and time spent for the visits)

2D	Arrange appointments and schedule doctors visits and lab tests

3	How would you rate the burden associated with taking care of paperwork from health insurance agencies, welfare organizations, hospitals and/or social care?

4	How would you rate the constraints associated with your diet (for example, not being allowed to eat certain foods)?

5	How would you rate the burden associated with the recommendations from your doctors to practice regular physical exercises?

6	What is the impact of your healthcare on your social relationships (for example, need for assistance, being ashamed to take your medication in front of people)?

7	'Frequent healthcare reminds me of my health problems'

**Figure 1:**
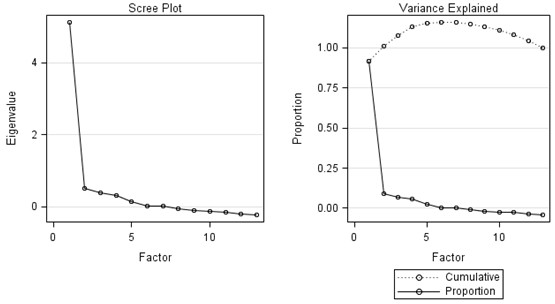
**Eigenvalue diagram of the factor analysis of the questionnaire for treatment burden**. The scree plot shows a break before factor 2, which suggests a unidimensional solution. 'Does not apply' was considered the lowest possible score (0).

Factorial validity, assessed by scree plots, favored a unidimensional instrument because 91% of the variance was explained by the first principal factor (Figure 1 and Additional file 3, Appendix 3). Cronbach's α was 0.89. The global score of the Treatment Burden Questionnaire was the sum of the answers to each item and ranged from 0 to 130. It was highly correlated with every item of the questionnaire (r_s _= 0.47 to 0.68) (Additional file 4, Appendix 4).

Construct validity showed (1) a moderate negative correlation of the Treatment Burden Questionnaire score with the TSQM global and convenience scores (r_S _= -0.41 and r_S _= -0.53) and a weak negative correlation with the TSQM efficacy score (r_S _= -0.26) (Table 3) and (2) a significant association of scores for variables used to describe treatment workload and the Treatment Burden Questionnaire global score (Table 4).

**Table 3:** Relationship between the Treatment Satisfaction Questionnaire for Medication (TSQM) scores and Treatment Burden Questionnaire global score (n = 502 patients)

	Correlation with the Treatment Burden Questionnaire global score^a^	*P *value
TSQM global score	-0.41	< 0.0001

TSQM efficacy score	-0.26	< 0.0001

TSQM convenience score	-0.53	< 0.0001

TSQM side effects score^a^	-0.52	< 0.0001

The TSQM assesses satisfaction with medication. Scores range from 0 to 100. A high score indicates high satisfaction with the medication. Negative coefficients indicate a decrease in the TSQM score associated with an increase in treatment burden.

^a^TSQM side effects score was calculated only for patients who declared experiencing side effects.

**Table 4:** Relationship between treatment workload variables and the Treatment Burden Questionnaire global score (n = 502 patients).

	Correlation with the Treatment Burden Questionnaire global score^a^	No. (%)	Mean score	*P *value
Correlation with treatment workload continuous variables				
Number of hospitalizations during the last year	0.24	-	-	< 0.0001
Number of medical appointments/month	0.28	-	-	< 0.0001
Number of different physicians	0.29	-	-	< 0.0001
Total number of tablets/day	0.25	-	-	< 0.0001
Total number of injections/day	0.31	-	-	< 0.0001
Number of drug intakes/day	0.16	-	-	0.0004
Time needed for healthcare per week (sum of the time needed for surveillance and the time needed to organize the treatment)	0.4	-	-	< 0.0001
Comparison between groups defined by treatment workload qualitative variables				
Need for a specific organization for daily care:				
Yes	**-**	338 (67.3%)	34.41	
No		164 (32.7%)	23.85	< 0.0001
Need for self-monitoring:				
Yes	**-**	168 (33.5%)	41.24	< 0.0001
No		334 (66.5%)	25.79	
Diet:				
Yes	-	198 (40.3%)	38.34	< 0.0001
No		293 (59.7%)	26.01	
Physical therapy:				
Yes	-	113 (22.9%)	35.77	
No		380 (77.1%)	29.73	0.02
Oxygen therapy:				
Yes	-	22 (4.4%)	44.68	
No		476 (94.8%)	30.47	0.005
Presence of side effects:	-			
Yes		168 (36.3%)	42.07	< 0.0001
No		295 (63.7%)	25.08	
The patient considers treatment as efficient:				
Yes	-	317 (63.1%)	27.35	< 0.0001
No		185 (36.8%)	37.13	

Spearman correlation coefficient for continuous variables and two-sided Wilcoxon two-sample test for qualitative variables

^a^Global score is the sum of all items scores of the questionnaire with 'Does not apply' and missing answers considered as the lowest possible score (0).

Using hierarchical ascendant classification, we clustered our sample in three homogenous groups of patients by the answers to the Treatment Burden Questionnaire (Additional file 5, Appendix 5). Twofold cross validation showed stable clustering results. The global score was 11.3 (± 9.2) in the first cluster, 34.6 (± 11.1) in the second cluster and 65.8 (± 18.1) in the third cluster. Therefore, we defined the clusters as patients with low, moderate and high burden of treatment. Descriptive analysis of the treatment workload items within the three clusters showed that scores for these variables were significantly higher for patients with high treatment burden (Table 5). Treatment workload variables could explain up to 69% of the variability in the patient's score. Prediction of global score with these variables was more accurate with high than low treatment burden (R^2 ^= 0.86 vs R^2 ^= 0.62) (Additional file 6, Appendix 6). Treatment burden score was significantly higher when patients experienced medication side effects (*P *< 0.0001) and for patients whose treatment did not relieve their symptoms (*P *< 0.0001).

**Table 5:** Characteristics of groups clustered by the hierarchical ascendant classification (n = 502 patients)

Characteristic	Whole sample (n = 502)	'Low burden' (n = 240)	'Moderate burden' (n = 140)	'High burden' (n = 122)	*P *value
Global score	30.1 ± 25.3	11.3 ± 9.2	34.6 ± 11.1	65.8 ± 18.1	< 0.0001
Age, years	59.3 ± 17.0	62.9 ± 16.1	59.2 ± 17.9	52.2 ± 15.6	< 0.0001
Female sex, no. (%)	266	118 (49.2%)	78 (55.7%)	70 (57.8%)	0.23
Marital status					0.19
Married	216	105 (45.3%)	60 (43.5%)	51 (42.9%)	
Live in partner	38	13 (5.6%)	12 (8.7%)	13 (10.9%)	
Single/separated	171	76 (32.8%)	49 (35.5%)	46 (38.7%)	
Widowed	64	38 (16.4%)	17 (12.3%)	9 (7.6%)	
Highest education level, no. (%)					0.76
No diploma/primary school	85	43 (19.9%)	23 (17.6%)	19 (17.1%)	
Secondary/high school	195	96 (44.4%)	54 (41.2%)	45 (40.5%)	
College	178	77 (35.6%)	54 (41.2%)	47 (42.3%)	
Inpatient, no. (percentage of the whole sample)	257	105 (43.7%)	84 (60.0%)	68 (55.7%)	0.004
Duration of disease, years	15.0 ± 15.4	16.3 ± 16.5	14.5 ± 16.7	13.3 ± 10.7	0.34
Presence of daily symptoms	300	110 (48.0%)	93 (70.4%)	97 (82.2%)	< 0.0001
Need for assistance, no. (%)	132	45 (18.8%)	41 (29.3%)	46 (38.0%)	0.0003
Number of hospitalizations during the last 12 months	1.1 ± 1.9	0.9 ± 1.8	1.2 ± 1.9	1.5 ± 2.1	0.0004
Number of medical appointments/month	2.3 ± 3.4	1.5 ± 1.6	2.6 ± 3.3	3.6 ± 5.2	< 0.0001
Number of different physicians	2.4 ± 1.4	2.1 ± 1.3	2.4 ± 1.3	2.9 ± 1.4	< 0.0001
Number of tablets/day	5.4 ± 4.5	4.5 ± 4.1	5.7 ± 3.9	7.0 ± 5.5	< 0.0001
Number of drug intakes/day	1.9 ± 1.0	1.8 ± 1.0	2.0 ± 1.0	2.1 ± 1.1	0.07
Number of injections/day	0.5 ± 1.3	0.2 ± 0.9	0.3 ± 0.9	1.3 ± 1.9	< 0.0001
Diet, no. (%)	198	75 (31.9%)	57 (42.2%)	66 (54.5%)	0.0002
Physical therapy, no. (%)	113	45 (19.2%)	33 (23.9%)	35 (28.9%)	0.11
Oxygen therapy, no. (%)	22	5 (2.1%)	8 (5.7%)	9 (7.4%)	0.05
Time needed to organize drugs/week*	22 ± 92 min	17 ± 100 min	13 ± 51 min	43 ± 108 min	< 0.0001
Need for self-monitoring, no. (%)	168	61 (25.4%)	36 (25.7%)	71 (58.2%)	< 0.0001
Time needed for self-monitoring/week*	14 ± 66 min	5 ± 31 min	10 ± 67 min	37 ± 101 min	< 0.0001
Presence of side effects, no. (%)	168	46 (20.9%)	56 (43.1%)	66 (58.4%)	< 0.0001
Patient considers his treatment efficient, no. (%)	317	176 (73.3%)	74 (52.9%)	67 (54.9%)	< 0.0001
Main chronic condition, no. (%)					< 0.0001
Diabetes	81	25 (10.7%)	14 (10.1%)	42 (35.0%)	
Rheumatologic diseases	59	25 (10.7%)	21 (15.2%)	13 (10.8%)	
Pulmonary diseases (other than asthma)	40	23 (9.9%)	12 (8.7%)	5 (4.2%)	
High blood pressure and dyslipidemia	44	29 (12.4%)	9 (6.5%)	6 (5.0%)	
Asthma	37	23 (9.9%)	8 (5.8%)	6 (5.0%)	
Systemic diseases	43	16 (6.9%)	13 (9.4%)	14 (11.7%)	

Patients were clustered in three groups depending on the similarity of their responses to the instrument. Global score was 11.3 (± 9.2) in the first cluster, 34.6 (± 11.1) in the second cluster and 65.8 (± 18.1) in the third cluster. Therefore, we defined the clusters as patients with low, moderate and high burden of treatment. Continuous variables are presented as mean ± SE. Categorical variables are presented as proportion of the corresponding subgroup. Associations between continuous variables among different classes were determined by Wilcoxon test. Qualitative variables are presented by their frequency in the whole sample. Associations between qualitative variables among different classes were determined by the χ^2 ^test. Global score is the sum of all items scores of the questionnaire with 'Does not apply' and missing answers considered the lowest possible score (0).

*Time needed for patients who did not require specific organization for daily care or who had no self-monitoring was considered 0.

**Figure 2:**
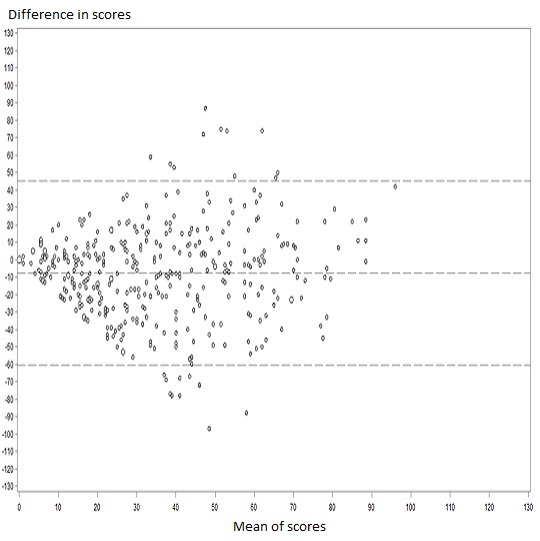
**Bland and Altman plot representing agreement between Treatment Burden Questionnaire global scores for patients and physicians (n = 396 patients)**. The difference between global score for patients and physicians is plotted against the mean score. Negative differences mean that physicians overestimated the burden and positive differences that they underestimated it. Horizontal lines are drawn at the mean difference between the two measurements and the upper and lower limits of agreement. The size of markers reflects the number of individual observations.

We found a moderate agreement (ICC 0.60 (0.28 to 0.79)) between patient and informal caregiver global scores (39 informal caregivers (7.8%) completed the questionnaire) (Additional file 7, Appendix 7a). Bland and Altman plots showed a mean difference of -8.7; 95% limits of agreement were -58.0 and 40.7 (Additional file 7, Appendix 7b). Agreement between patient and physician global scores was weak (ICC 0.38 (0.29 to 0.47)) (396 physicians (78.9%) completed the questionnaire) (Additional file 8, Appendix 8). Bland and Altman plot showed a mean difference of -7.6; 95% limits of agreement were -60.7 and 45.4 (Figure 2). Agreement between patient and general practitioner (n = 209) evaluations was ICC = 0.42 (0.27 to 0.54). Agreement between patient and hospital specialists (n = 187) evaluations was ICC = 0.29 (0.14 to 0.42) (Additional file 9, Appendix 9). Treatment workload variables could explain up to 76% of the variation in physician evaluations and was more accurate for patients with high than low treatment burden (R^2 ^= 0.82 vs R^2 ^= 0.72) (Additional file 6, Appendix 6).

Retests were obtained for 211 patients (42.0%). For the global score, the ICC for all retests was 0.76 (0.67 to 0.83) (Additional file 10, Appendix 10a). Bland and Altman plots showed a mean difference of -5.9; 95% limits of agreement were -42.4 and 30.5 (Additional file 10, Appendix 10b). Reliability for the 2-week retest group (n = 182) was consistent with the 1-month retest group (n = 29) (ICC = 0.75 (0.65 to 0.83) vs ICC = 0.78 (0.46 to 0.91)).

## Discussion

In this study, we presented a unidimensional valid and reliable instrument assessing the treatment burden of chronic diseases for patients with multiple chronic conditions. This patient-reported measure took into account the burden associated with drug intake, surveillance, lifestyle changes and the impact of healthcare on social relationships.

The instrument could help in clinical research for developing clinical practice guidelines adapted to the realities of patient lives. In addition, it could be used in clinical practice as a validated global score that is easy to calculate to identify patients overwhelmed by their treatment to help begin conversations about treatment burden with these patients.

We highlighted a negative correlation between treatment burden and treatment satisfaction: the more satisfied patients were with their treatment, the less the treatment burden. We expected that our scale score would correlate highly with the TSQM convenience score because some items overlapped. However, patients with side effects and who found the treatment inefficient would feel less agreeable to integrate the treatment in their lives.

Treatment burden did not concern only patients taking a lot of medications: 25% of patients in our sample took < 3 medications a day and still had a median treatment burden score of 17 (Q1 to Q3: 6 to 36). Therefore, treatment burden should be taken into account for every patient, because it could be associated with adherence to care [9] and thus could contribute to hospitalizations and survival rates [11]. However, physicians were often not fully aware of their patients' investment of time and efforts to comply with every prescription: we found only weak agreement between evaluation of treatment burden between patients and physicians. Even for specific domains such as self-monitoring or the prescription of a diet, physicians could not predict their patient's evaluation. General practitioners, who are coordinators of care in France, have better knowledge than hospital specialists of how patients cope with everything they do to take care of their health (ICC = 0.42 for general practitioners and 0.20 for hospital specialists) but still fail to assess patients' treatment burden accurately. This finding is not unexpected, because treatment burden is a relatively new concept to physicians [13] and expresses a patient experience that is not shared in depth during consultations [36].

In existing questionnaires, treatment burden was often considered only as a subscale for larger disease-specific scales [16,17] and focused on a single treatment regimen. Given the increasing number of patients with multiple chronic diseases and complex treatment regimens, measuring global treatment burden seems increasingly important. As Gallacher *et al. *have shown for chronic heart failure, treatment burden relates to how patients cope with their treatment [37]: (1) learning about treatment and their consequences, (2) monitoring the treatment, (3) adhering to treatment and lifestyle changes and (4) engaging with others. During our study, we asked patients about aspects of their healthcare that were not mentioned in our questionnaire but had an impact on their lives. We found the same domains of treatment burden as Gallacher *et al.*, with the exception of gaining an understanding about illness and treatments. Nevertheless, acquiring this knowledge is an important burden in the management of chronic conditions, especially when patients have to make sense of the disparate and conflicting information they gather from different sources. However, because we recruited patients with illnesses for at least 6 months, they might have already coped with this particular burden, adapted to it, and therefore did not mention it.

The strengths of this study included field testing the instrument in a large sample of both inpatients and outpatients with different conditions and treatment regimens, which ensured that our instrument was flexible enough for assessing the treatment burden across any disease or context. However, we found a significant floor effect and a large proportion of 'Does not apply' responses for all of our scales. This result was expected because treatment burden depends on how patients cope with their treatment regimens. Therefore, patients could have no burden in aspects of their care they have integrated in their lives. As well, patients with similar treatment regimens could have very different treatment burdens. Still, domains not included in this instrument may be critical to some of these patients. During the validation study, we systematically searched for other aspects of treatment burden that could have an impact on patients' quality of life but found no preeminent domain.

More work in measuring treatment burden is needed. Because treatment burden depends on the context of patients (social or family structure, care delivery system) [13] and because our instrument was developed in France, we could not exclude that different domains could arise in other settings. As an example, the financial burden of the treatment did not arise from our qualitative interviews because the public health insurance program in France guarantees healthcare free of charge for patients with chronic conditions. In addition, depending on the social or family structure, the treatment burden may be shared by the patient with one or more informal caregivers, thus affecting the validity of the measure when only reported by the patient.

## Conclusions

Our instrument on treatment burden for patients exhibiting multiple chronic conditions provides the first valid and reliable solution to assess the burden of treatment across any disease or treatment context. It may help in the development of treatment strategies that are both efficient and acceptable for patients.

## Competing interests

The authors declare that they have no competing interests.

## Authors' contributions

V-TT, BF and PR conceived and designed the study. V-TT and DB acquired the data. V-TT, BF, PR analyzed and interpreted the data. V-TT and PR drafted the manuscript. VM, BF, DE, DB and PR critically revised the manuscript for important intellectual content. DB and PR provided administrative, technical, and material support. All authors saw and approved the final manuscript. PR is the guarantor, had full access to the data in the study, and takes responsibility for the integrity of the data and the accuracy of the data analysis.

## Pre-publication history

The pre-publication history for this paper can be accessed here:

http://www.biomedcentral.com/1741-7015/10/68/prepub

## Supplementary Material

Additional file 1 **Appendix 1**. Demographic and clinical characteristics of patients included in the semistructured interview pretest (n = 22). Click here for file

Additional file 2 **Appendix 2**. Characteristics of the items presented to patients (n = 502 patients). Click here for file

Additional file 3 **Appendix 3**. Eigenvalues for the correlation matrix. Click here for file

Additional file 4 **Appendix 4**. Association of items of the Treatment Burden Questionnaire and global score (n = 502 patients). Click here for file

Additional file 5 **Appendix 5**. Dendogram of the hierarchical ascendant classification of patients by their answers to the Treatment Burden Questionnaire (n = 502 patients). Click here for file

Additional file 6 **Appendix 6**. (a). Linear regression analysis of relation between global score for Treatment Burden Questionnaire as assessed by patients with variables associated with treatment workload. (b) Linear regression analysis of relation between global score for Treatment Burden Questionnaire as assessed by physicians with variables associated with treatment workload. Click here for file

Additional file 7 **Appendix 7**. (a) Agreement between patients and informal caregiver evaluations of the treatment burden (n = 39). (b) Bland and Altman plot representing agreement between Treatment Burden Questionnaire global scores for patients and informal caregivers (n = 39). Click here for file

Additional file 8 **Appendix 8**. Agreement between patients and physician evaluations of the treatment burden (n = 396). Click here for file

Additional file 9 **Appendix 9**. Validation of the instrument in different subgroups. Click here for file

Additional file 10 **Appendix 10**. (a) Reliability using test-retest (n = 211). (b) Bland and Altman plot representing the test-retest reliability of the Treatment Burden Questionnaire global score (n = 211). Click here for file
