# Supplementary material for: Development and description of measurement properties of an instrument to assess treatment burden among patients with multiple chronic conditions
Source: BMC Med. 2012 Jul 4;10:68. doi: 10.1186/1741-7015-10-68 (PMC3402984; doi:10.1186/1741-7015-10-68)
Supplement: Additional file 1 — Appendix 1. Demographic and clinical characteristics of patients included in the semistructured interview pretest (n = 22). [file 1741-7015-10-68-S1.DOCX]

| Characteristic | Value |
| --- | --- |
| Age – yr | 70 (53-76) |
| Female sex – no. (%) | 11 (50.0%) |
| Marital status – no. (%)  Married  Live in partner  Single/separated  Widowed | 11 (50.0 %)  2 (9.1 %)  3 (13.6 %)  6 (27.3 %) |
| Highest education level –no. (%)  No diploma /Primary school  Secondary / High school  College | 7 (33.3 %)  4 (19.0 %)  10 (47.7 %) |
| Inpatient – no. (%) | 6 (27.3 %) |
| Duration of disease – yr | 11 (5-23) |
| Presence of daily symptoms – no. (%) | 18 (85.7 %) |
| Need of assistance – no. (%) | 7 (31.8 %) |
| Number of hospitalization during the last 12 months | 0 (0-3) |
| Number of medical appointments/month | 1 (0-3) |
| Number of different physicians | 3 (2-4) |
| Number of tablets/day | 5 (4-6) |
| Number of drug intakes/day | 2 (2-2) |
| Number of injections/day | 0 (0-0) |
| Diet – no. (%) | 13 (59.1 %) |
| Physical therapy – no. (%) | 6 (27.1 %) |
| Oxygen therapy – no. (%) | 5 (22.0 %) |

Appendix 1. Demographic and clinical Characteristics of patients included in the pretest (n=22). Median (range) were reported for continuous variables and number (%) for categorical variables.
