# Supplementary material for: Development and description of measurement properties of an instrument to assess treatment burden among patients with multiple chronic conditions
Source: BMC Med. 2012 Jul 4;10:68. doi: 10.1186/1741-7015-10-68 (PMC3402984; doi:10.1186/1741-7015-10-68)
Supplement: Additional file 2 — Appendix 2. Characteristics of the items presented to patients (n = 502 patients). [file 1741-7015-10-68-S2.DOCX]

| Item | Response rate  No. (%) | Item  “Does not apply”  No. (%) | Floor effect  No. (%) | “High burden” (score>7)  No. (%) |
| --- | --- | --- | --- | --- |
| 1A. The taste, shape or size of your tablets and/or the inconvenience caused by your injections (e.g., pain, bleeding, scars) | 496  (98.8 %) | 91  (18.3 %) | 180  (44.4 %) | 44  (10.9 %) |
| 1B. The number of times you have to take your medication every day | 498  (99.2 %) | 61  (12.2 %) | 166  (38.0 %) | 36  (8.2 %) |
| 1C. The things you do to remind yourself to take your daily medication and/or to manage your treatment when you are not at home. | 499  (99.4 %) | 71  (14.2 %) | 149  (34.8 %) | 60  (14.0 %) |
| 1D. The specific conditions when taking your medication (e.g., taking it at a specific time of the day or meal, not being able to do certain things after taking them like driving or lying down) | 501  (99.8 %) | 132  (26.3 %) | 166  (45.0 %) | 45  (12.2 %) |
| 1E. The conditions for storing your medications (e.g., in your refrigerator) | 500  (99.6 %) | 258  (51.6 %) | 155  (64.0 %) | 29  (12.0 %) |
| 2A. Lab tests and other exams (frequency, time spent and inconvenience of these exams) | 500  (99.6 %) | 31  (6.2 %) | 104  (22.2 %) | 85  (18.1 %) |
| 2B. Self-monitoring (e.g., taking your blood pressure or measuring your blood sugar yourself: frequency, time spent and inconvenience of this surveillance) | 499  (99.4 %) | 243  (48.7 %) | 91  (35.5 %) | 43  (16.8 %) |
| 2C. Doctors visits (frequency and time spent for the visits) | 496  (98.8 %) | 27  (5.4 %) | 126  (26.9 %) | 75  (16.0 %) |
| 2D. Arrange appointments and schedule doctors visits and lab tests | 499  (99.4 %) | 35  (7.0 %) | 129  (27.8 %) | 87  (18.7 %) |
| 3. How would you rate the burden associated with taking care of paperwork from health insurance agencies, welfare organizations, hospitals and/or social care? | 499  (99.4 %) | 56  (11.2 %) | 146  (33.0 %) | 84  (19.0 %) |
| 4. How would you rate the constraints associated with your diet (e.g., not being allowed to eat certain food)? | 500  (99.6 %) | 183  (36.6 %) | 87  (27.4 %) | 73  (23.0 %) |
| 5. How would you rate the burden associated with the recommendations from your doctors to practice regular physical exercises? | 496  (98.8 %) | 138  (27.8 %) | 128  (35.7 %) | 73  (20.4 %) |
| 6. What is the impact of your healthcare on your social relationships (e.g., need for assistance, being ashamed to take your medication in front of people)? | 498  (99.2 %) | 84  (16.9 %) | 218  (52.7 %) | 67  (16.2 %) |
| 7. "Frequent healthcare reminds me of my health problems" | 495  (98.6 %) | - | 187  (37.8 %) | 128  (22.2 %) |

Appendix 2. Characteristics of the items presented to patients (n=502 patients). Floor-effect frequencies and “high burden patients” frequencies relate to patients concerned by the item and thus not checking “Does not apply”.
