# Supplementary material for: Development and description of measurement properties of an instrument to assess treatment burden among patients with multiple chronic conditions
Source: BMC Med. 2012 Jul 4;10:68. doi: 10.1186/1741-7015-10-68 (PMC3402984; doi:10.1186/1741-7015-10-68)
Supplement: Additional file 3 — Appendix 3. Eigenvalues for the correlation matrix. [file 1741-7015-10-68-S3.DOCX]

Appendix 3. Eigenvalues for the correlation matrix. “Does not apply” were considered as the lowest possible score (0).

|  | Eigenvalue | Proportion of variance | Cumulative variance |
| --- | --- | --- | --- |
| 1 | 5.12 | 0.92 | 0.92 |
| 2 | 0.50 | 0.09 | 1.00 |
