# Supplementary material for: Development and description of measurement properties of an instrument to assess treatment burden among patients with multiple chronic conditions
Source: BMC Med. 2012 Jul 4;10:68. doi: 10.1186/1741-7015-10-68 (PMC3402984; doi:10.1186/1741-7015-10-68)
Supplement: Additional file 4 — Appendix 4. Association of items of the Treatment Burden Questionnaire and global score (n = 502 patients). [file 1741-7015-10-68-S4.DOCX]

Appendix 4. Association of items of the Treatment Burden Questionnaire and global score (n=502 patients). Correlation of each individual item with the scale total, omitting that item from the total, by Spearman correlation coefficient.

| Item | Spearman correlation coefficient |
| --- | --- |
| 1A. The taste, shape or size of your tablets and/or the inconvenience caused by your injections (e.g., pain, bleeding, scars) | 0.60 (p<0.0001) |
| 1B. The number of times you have to take your medication every day | 0.61 (p<0.0001) |
| 1C. The things you do to remind yourself to take your daily medication and/or to manage your treatment when you are not at home. | 0.54 (p<0.0001) |
| 1D. The specific conditions when taking your medication (e.g., taking it at a specific time of the day or meal, not being able to do certain things after taking them like driving or lying down) | 0.58 (p<0.0001) |
| 2A. Lab tests and other exams (frequency, time spent and inconvenience of these exams) | 0.68 (p<0.0001) |
| 2B. Self-monitoring (e.g., taking your blood pressure or measuring your blood sugar yourself: frequency, time spent and inconvenience of this surveillance) | 0.54 (p<0.0001) |
| 2C. Doctors visits (frequency and time spent for the visits) | 0.67 (p<0.0001) |
| 2D. Arrange appointments and schedule doctors visits and lab tests | 0.68 (p<0.0001) |
| 3. How would you rate the burden associated with taking care of paperwork from health insurance agencies, welfare organizations, hospitals and/or social care? | 0.55 (p<0.0001) |
| 4. How would you rate the constraints associated with your diet (e.g., not being allowed to eat certain food)? | 0.47 (p<0.0001) |
| 5. How would you rate the burden associated with the recommendations from your doctors to practice regular physical exercises? | 0.47 (p<0.0001) |
| 6. What is the impact of your healthcare on your social relationships (e.g., need for assistance, being ashamed to take your medication in front of people)? | 0.52 (p<0.0001) |
| 7. "Frequent healthcare reminds me of my health problems" | 0.57 (p<0.0001) |
