# Supplementary material for: Development and description of measurement properties of an instrument to assess treatment burden among patients with multiple chronic conditions
Source: BMC Med. 2012 Jul 4;10:68. doi: 10.1186/1741-7015-10-68 (PMC3402984; doi:10.1186/1741-7015-10-68)

Appendix 5. Dendogram of the hierarchical ascendant classification of patients by their answers to the Treatment Burden Questionnaire (n=502 patients). Hierarchical ascendant classification was performed using a Ward's distance method. Classification resulted in the formation of 3 clusters of patients which could be described as patients with low burden of treatment, medium burden of treatment and high burden of treatment.


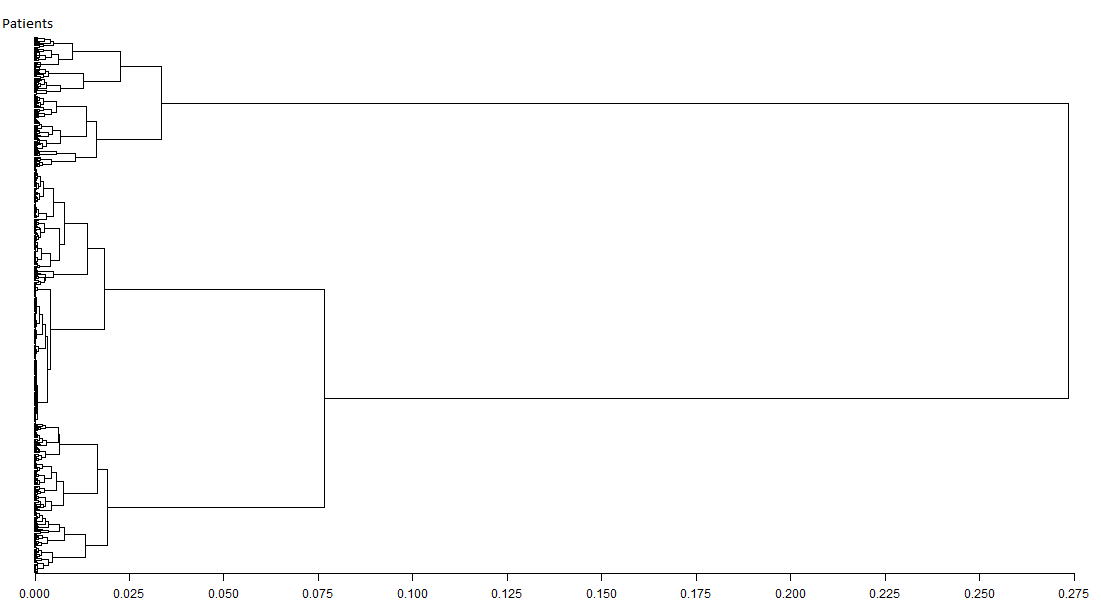

Supplement: Additional file 5 — Appendix 5. Dendogram of the hierarchical ascendant classification of patients by their answers to the Treatment Burden Questionnaire (n = 502 patients). [file 1741-7015-10-68-S5.DOCX]
