# Supplementary material for: Development and description of measurement properties of an instrument to assess treatment burden among patients with multiple chronic conditions
Source: BMC Med. 2012 Jul 4;10:68. doi: 10.1186/1741-7015-10-68 (PMC3402984; doi:10.1186/1741-7015-10-68)
Supplement: Additional file 6 — Appendix 6. (a). Linear regression analysis of relation between global score for Treatment Burden Questionnaire as assessed by patients with variables associated with treatment workload. (b) Linear regression analysis of relation between global score for Treatment Burden Questionnaire as assessed by physicians with variables associated with treatment workload. [file 1741-7015-10-68-S6.DOCX]

Appendix 6(a). Linear regression analysis of relation between global score for Treatment Burden Questionnaire as assessed by patients with variables associated with treatment workload

Treatment workload variables significantly associated with global score in the whole sample (n=502 patients). R²=0.69

|  | β | SE | p |
| --- | --- | --- | --- |
| Number of hospitalizations | 1.69 | 0.68 | 0.01 |
| Number of different physicians | 4.19 | 0.68 | <0.0001 |
| Number of medical appointments/month | 1.92 | 0.31 | <0.0001 |
| Number of tablets/day | 1.28 | 0.28 | <0.0001 |
| Number of injections/day | 5.30 | 1.21 | <0.0001 |
| Diet | 6.05 | 2.23 | 0.007 |
| Need of self monitoring | 6.21 | 2.73 | 0.02 |

Treatment workload variables significantly associated with global score for patients with low burden of treatment as defined by the hierarchical ascendant classification (HAC) (n=240 patients). R²=0.62

|  | β | SE | P |
| --- | --- | --- | --- |
| Number of different physicians | 2.78 | 0.39 | <0.0001 |
| Number of medical appointments/month | 1.90 | 0.40 | <0.0001 |
| Diet | 4.97 | 1.34 | 0.0003 |

Treatment workload variables significantly associated with global score for patients with moderate burden of treatment as defined by the hierarchical ascendant classification (HAC) (n=140 patients). R²=0.77

|  | β | SE | P |
| --- | --- | --- | --- |
| Number of hospitalizations | 2.19 | 0.94 | 0.02 |
| Number of different physicians | 5.96 | 0.92 | <0.0001 |
| Number of tablets/day | 1.72 | 0.39 | <0.0001 |
| Diet | 9.48 | 3.23 | 0.004 |

Treatment workload variables significantly associated with the global score for patients with high burden of treatment as defined by the hierrchical ascendant classification (HAC) (n=122 patients). R²=0.86

|  | β | SE | p |
| --- | --- | --- | --- |
| Number of different physicians | 11.68 | 1.46 | <0.0001 |
| Number of tablets/day | 2.32 | 0.55 | <0.0001 |
| Number of injections/day | 4.36 | 1.89 | 0.02 |
| Need of self monitoring | 12.93 | 5.43 | 0.02 |

Appendix 6(b). Linear regression analysis of relation between global score for Treatment Burden Questionnaire as assessed by physicians with variables associated with treatment workload

Treatment workload variables significantly associated with the physician’s global score in the whole sample (n=502). R²=0.76

|  | β | SE | p |
| --- | --- | --- | --- |
| Number of different physicians | 5.57 | 0.84 | <0.0001 |
| Number of tablets/day | 1.79 | 0.28 | <0.0001 |
| Number of injections/day | 4.14 | 1.26 | 0.001 |
| Diet | 9.21 | 3.00 | 0.002 |
| Need for self-monitoring | 15.39 | 3.25 | <0.0001 |

Treatment workload variables significantly associated with the physician’s global score for patients with a low burden of treatment as defined by the hierarchical ascendant classification (HAC) (n=240). R²=0.72

|  | β | SE | p |
| --- | --- | --- | --- |
| Number of different physicians | 5.53 | 1.16 | <0.0001 |
| Number of tablets/day | 1.42 | 0.39 | 0.0005 |
| Number of injections/day | 4.81 | 2.41 | 0.05 |
| Diet | 9.43 | 4.43 | 0.03 |
| Prescription of oxygen | 24.37 | 10.42 | 0.02 |
| Need for self-monitoring | 13.40 | 4.71 | 0.005 |

Treatment workload variables significantly associated with the physician’s global score for patients with a moderate burden of treatment as defined by the hierarchical ascendant classification (HAC) (n=140). R²=0.74

|  | β | SE | p |
| --- | --- | --- | --- |
| Number of different physicians | 3.84 | 1.69 | 0.03 |
| Number of tablets/day | 2.72 | 0.61 | <0.0001 |
| Number of injections/day | 8.15 | 3.21 | 0.01 |
| Diet | 14.77 | 5.58 | 0.01 |

Treatment workload variables significantly associated with the physician’s global score for patients with a high burden of treatment as defined by the hierarchical ascendant classification (HAC) (n=122). R²=0.82

|  | β | SE | p |
| --- | --- | --- | --- |
| Number of different physicians | 8.67 | 1.51 | <0.0001 |
| Number of tablets/day | 1.16 | 0.51 | 0.03 |
| Need for self-monitoring | 26.58 | 5.14 | <0.0001 |
