# Supplementary material for: Development and description of measurement properties of an instrument to assess treatment burden among patients with multiple chronic conditions
Source: BMC Med. 2012 Jul 4;10:68. doi: 10.1186/1741-7015-10-68 (PMC3402984; doi:10.1186/1741-7015-10-68)

| Item | ICC | 95% CI |
| --- | --- | --- |
| 1A. The taste, shape or size of your tablets and/or the inconvenience caused by your injections (e.g., pain, bleeding, scars) | 0.28 | [0-0.60] |
| 1B. The number of times you have to take your medication every day | 0.58 | [0.25-0.80] |
| 1C. The things you do to remind yourself to take your daily medication and/or to manage your treatment when you are not at home. | 0.59 | [0.34-0.80] |
| 1D. The specific conditions when taking your medication (e.g., taking it at a specific time of the day or meal, not being able to do certain things after taking them like driving or lying down) | 0.50 | [0.16-0.78] |
| 2A. Lab tests and other exams (frequency, time spent and inconvenience of these exams) | 0.41 | [0-0.70] |
| 2B. Self-monitoring (e.g., taking your blood pressure or measuring your blood sugar yourself: frequency, time spent and inconvenience of this surveillance) | 0.46 | [0.02-0.77] |
| 2C. Doctors visits (frequency and time spent for the visits) | 0.29 | [0-0.60] |
| 2D. Arrange appointments and schedule doctors visits and lab tests | 0.44 | [0.08-0.69] |
| 3. How would you rate the burden associated with taking care of paperwork from health insurance agencies, welfare organizations, hospitals and/or social care? | 0.47 | [0.17-0.75] |
| 4. How would you rate the constraints associated with your diet (e.g., not being allowed to eat certain food)? | 0.53 | [0.15-0.82] |
| 5. How would you rate the burden associated with the recommendations from your doctors to practice regular physical exercises? | 0.47 | [0.09-0.76] |
| 6. What is the impact of your healthcare on your social relationships (e.g., need for assistance, being ashamed to take your medication in front of people)? | 0.41 | [0.13-0.70] |
| 7. "Frequent healthcare reminds me of my health problems" | 0.48 | [0.17-0.72] |

Appendix 7(a). Agreement between patients and informal caregiver evaluations of the treatment burden (n=39) with ICC for agreement. 95% confidence intervals (95% CIs) were calculated by a bootstrap method. *Global score is the sum of all item scores with “Does not apply” and missing answers having the lowest possible score (0).

Appendix 7(b). Bland and Altman plot representing agreement between Treatment Burden Questionnaire global scores for patients and informal caregivers (n=39). The difference between patient and informal caregiver scores is plotted against the mean score. Negative differences indicate that informal caregivers overestimated the burden and positive differences that they underestimated it. Horizontal lines are drawn for the mean difference between the two measurements and the upper and lower limits of agreement.


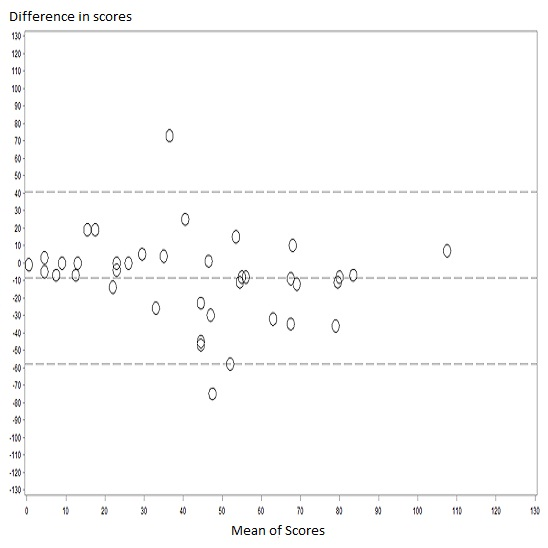

Supplement: Additional file 7 — Appendix 7. (a) Agreement between patients and informal caregiver evaluations of the treatment burden (n = 39). (b) Bland and Altman plot representing agreement between Treatment Burden Questionnaire global scores for patients and informal caregivers (n = 39). [file 1741-7015-10-68-S7.DOCX]
