# Supplementary material for: Development and description of measurement properties of an instrument to assess treatment burden among patients with multiple chronic conditions
Source: BMC Med. 2012 Jul 4;10:68. doi: 10.1186/1741-7015-10-68 (PMC3402984; doi:10.1186/1741-7015-10-68)
Supplement: Additional file 8 — Appendix 8. Agreement between patients and physician evaluations of the treatment burden (n = 396). [file 1741-7015-10-68-S8.DOCX]

| Item | ICC | 95% CI |
| --- | --- | --- |
| 1A. The taste, shape or size of your tablets and/or the inconvenience caused by your injections (e.g., pain, bleeding, scars) | 0.23 | [0.13-0.33] |
| 1B. The number of times you have to take your medication every day | 0.25 | [0.15-0.35] |
| 1C. The things you do to remind yourself to take your daily medication and/or to manage your treatment when you are not at home. | 0.23 | [0.14-0.33] |
| 1D. The specific conditions when taking your medication (e.g., taking it at a specific time of the day or meal, not being able to do certain things after taking them like driving or to lying down) | 0.22 | [0.12-0.34] |
| 2A. Lab tests and other exams (frequency, time spent and inconvenience of these exams) | 0.30 | [0.19-0.39] |
| 2B. Self-monitoring (e.g., taking your blood pressure or measuring your blood sugar yourself: frequency, time spent and inconvenience of this surveillance) | 0.41 | [0.30-0.51] |
| 2C. Doctors visits (frequency and time spent for the visits) | 0.24 | [0.15-0.34] |
| 2D. Arrange appointments and schedule doctors visits and lab tests | 0.22 | [0.12-0.31] |
| 3. How would you rate the burden associated with taking care of paperwork from health insurance agencies, welfare organizations, hospitals and/or social care? | 0.28 | [0.18-0.38] |
| 4. How would you rate the constraints associated with your diet (e.g., not being allowed to eat certain food)? | 0.38 | [0.28-0.47] |
| 5. How would you rate the burden associated with the recommendations from your doctors to practice regular physical exercises? | 0.19 | [0.09-0.29] |
| 6. What is the impact of your healthcare on your social relationships (e.g., need for assistance, being ashamed to take your medication in front of people)? | 0.20 | [0.11-0.31] |
| 7. "Frequent healthcare reminds me of my health problems" | 0.21 | [0.11-0.29] |
| Global score* | 0.38 | [0.29-0.47] |

Appendix 8. Agreement between patients and physician evaluations of the treatment burden (n=396) with ICC for agreement. 95% CIs were calculated by a bootstrap method. *Global score is the sum of all item scores with “Does not apply” and missing answers having the lowest possible score (0).
