# Supplementary material for: Development and description of measurement properties of an instrument to assess treatment burden among patients with multiple chronic conditions
Source: BMC Med. 2012 Jul 4;10:68. doi: 10.1186/1741-7015-10-68 (PMC3402984; doi:10.1186/1741-7015-10-68)
Supplement: Additional file 9 — Appendix 9. Validation of the instrument in different subgroups. [file 1741-7015-10-68-S9.DOCX]

|  | Global score | p | Agreement between the physician and the patient | Correlation with TSQM scores using Spearman correlation coefficient | |
| --- | --- | --- | --- | --- | --- |
|  |  |  |  | TSQM global score | TSQM convenience score |
| Age  <60 (n= 243)  >60 (n= 259) | 38.4 (**±** 26.7)  24.0 (**±** 21.9) | <0.0001 | 0.36 [0.25-0.49]  0.37 [0.23-0.47] | -0.39 (p<0.0001)  -0.36 (p<0.0001) | -0.53 (p<0.0001)  -0.47 (p<0.0001) |
| Presence of symptoms  Symptomatic (n= 300)  Asymptomatic (n= 179 ) | 37.6 (**±** 26.0)  20.6 (**±** 20.7) | <0.0001 | 0.41 [0.28-0.51]  0.27 [0.14-0.42] | -0.32 (p<0.0001)  -0.34 (p<0.0001) | -0.46 (p<0.0001)  -0.50 (p<0.0001) |
| Educational level  No diploma/ Primary (n= 85)  Secondary/ high school (n= 195)  College (n= 178) | 29.0 (**±** 26.9)  29.1 (**±** 24.5)  34.3 (**±** 25.3) | 0.04 | 0.54 [0.17-0.69]  0.35 [0.21-0.48]  0.36 [0.18-0.51] | -0.37 (p=0.0008)  -0.44 (p<0.0001)  -0.41 (p<0.0001) | -0.56 (p<0.0001)  -0.50 (p<0.0001)  -0.57 (p<0.0001) |
| Inpatients (n=257)  Outpatients (n= 245) | 34.7(**±** 27.7)  27.1(**±** 23.1) | 0.001 | 0.29 [0.14-0.42]  0.42 [0.27-0.54] | -0.36 (p<0.0001)  -0.45 (<0.0001) | -0.50 (p<0.0001)  -0.56 (p<0.0001) |
| Different conditions reported as “main chronic condition”  Diabetes (n=81)  Rheumatologic diseases (n=59)  High blood pressure and dyslipidemia (n=44)  Systemic diseases (n=43)  Pulmonary diseases (other than asthma) (n=40)  Heart diseases (n=37) | 46.4 (**±** 28.6)  28.6 (**±** 26.3)  18.5 (**±** 19.9)  39.0 (**±** 26*.*3)  24.8 (**±** 17.5)  29.3 (**±** 23.7) | <0.0001 | 0.23 [0-0.44]  0.08 [0-0.42]  0.38 [0.12-0.74]  0.15 [0-0.46]  0.40 [0.10-0.64]  0.39 [0.07-0.67] | -0.28 (p=0.01)  -0.41 (p=0.001)  -0.32 (p=0.04)  -0.34 (p=0.02)  -0.55 (p=0.0003)  -0.17 (p=0.33) | -0.55 (p<0.0001)  -0.49 (p<0.0001)  -0.38 (p=0.01)  -0.35 (p=0.02)  -0.56 (p=0.0003)  -0.55 (p=0.0005) |

Appendix 9. Validation of the instrument in different subgroups. Global score is presented as mean (± SD). Wilcoxon and Kruskal Wallis tests were used for comparing subgroups. Agreement between physician and patient measurements, with ICC for agreement and a bootstrap method for calculating 95% CIs, was consistent with results obtained for the whole sample except for rheumatologic diseases, where it was weaker. Spearman correlation coefficient was used to assess the correlations between the global score and the TSQM scores.
